# Cost-effectiveness of diagnosing and treating patients with early Alzheimer's disease with anti-amyloid treatment in a clinical setting

**DOI:** 10.1177/13872877251323231

**Published:** 2025-03-20

**Authors:** Anders Wimo, Ron Handels, Kaj Blennow, Bjørn-Eivind Kirsebom, Per Selnes, Jaka Bon, Andreja Emersic, Fernando Gonzalez-Ortiz, Milica Gregoric Kramberger, Anders Sköldunger, Andreja Speh, Santiago Timón-Reina, Ellen Vromen, Pieter Jelle Visser, Bengt Winblad, Tormod Fladby

**Affiliations:** 1 Department of Neurobiology, Care Sciences and Society, Division of Neurogeriatrics, BioClinicum J9:20, Karolinska Institutet, Solna, Sweden; 2 Maastricht University, Alzheimer Centre Limburg, Faculty of Health, Medicine and Life Sciences, Maastricht, Netherlands; 3 Department of Psychiatry and Neuropsychology, School for Mental Health and Neuroscience, Maastricht, The Netherlands; 4 Department of Psychiatry and Neurochemistry, Institute of Neuroscience and Physiology, The Sahlgrenska Academy, University of Gothenburg, Gothenburg, Sweden; 5 Clinical Neurochemistry Laboratory, Sahlgrenska University Hospital, Mölndal, Sweden; 6 Department of Neurology, University Hospital of North Norway, Tromsø, Norway; 7 Department of Psychology, Faculty of Health Sciences, The Arctic University of Norway, Tromsø, Norway; 8 Department of Neurology, Akershus University Hospital, Lørenskog, Norway; 9 Institute of Clinical Medicine, University of Oslo, Oslo, Norway; 10 Department of Research, Akershus University Hospital, Lørenskog, Norway; 11 Department of Neurology, University Medical Centre Ljubljana, Ljubljana, Slovenia; 12 Faculty of Pharmacy, University of Ljubljana, Ljubljana, Slovenia; 13 Medical Faculty, University of Ljubljana, Ljubljana, Slovenia; 14 Department of Nursing, Biologihuset, Umeå University, Umeå, Sweden; 15 Alzheimer Center Amsterdam, Neurology, Vrije Universiteit Amsterdam, Amsterdam UMC Location Vumc, Amsterdam, the Netherlands; 16 Amsterdam Neuroscience, Neurodegeneration, Amsterdam, The Netherlands; 17 Department of Psychiatry, Maastricht University, Maastricht, the Netherlands; 18 Karolinska University Hospital, Theme Inflammation and Aging, Stockholm, Sweden

**Keywords:** Alzheimer's disease, anti-amyloid treatment, biomarkers, blood-based biomarkers, cost-effectiveness, disease modifying treatment, donanemab, lecanemab

## Abstract

**Background:**

The introduction of anti-amyloid treatments (AAT) for Alzheimer's disease (AD) has put the cost-effectiveness into focus.

**Objective:**

Estimate the potential cost-effectiveness of diagnostic pathways combined with AAT for early AD.

**Methods:**

Diagnostic accuracy of blood-based (BBM) and cerebrospinal fluid (CSF) biomarkers was obtained from Norwegian memory clinics using positron emission tomography (PET) as reference standard. In a health-economic model, the cost-effectiveness of three diagnostic strategies was estimated relying either on BBM (p-tau 217), CSF (Aβ_42/40_ ratio), and BBM with CSF confirmatory testing and compared with standard of care (SoC) and compared with CSF-AAT. The model consisted of a decision tree reflecting the diagnostic process and a subsequent Markov cohort model starting at mild cognitive impairment due to AD. All strategies except SoC were combined with AAT including costs of treatment (assumed €5000/year), infusions and monitoring.

**Results:**

Compared with SoC all three strategies (CSF-AAT, BBM-AAT, and BBM-CSF-AAT) resulted in QALY gains at higher costs, with an incremental cost-effectiveness ratio (ICER) of 110k€, 141k€ and 110k€ respectively. Compared with CSF-AAT both BBM-AAT and BBM-CSF-AAT strategies resulted in QALYs lost at lower costs, with an ICER of 27k€ and 109k€ respectively. Results were particularly sensitive to the price of AAT and possible subcutaneous administration.

**Conclusions:**

Compared with SoC all three strategies are potentially not cost-effective as they exceeded the Swedish maximum willingness to pay threshold of €94,800 per QALY gained. BBM-CSF-AAT versus CSF-AAT is potentially cost-effective if willing to accept its QALY loss. Discussions on budget impact on different payers are needed after introducing AAT.

## Introduction

Alzheimer's disease (AD) is a progressive brain disorder causing the majority of dementia cases.^1^ AD slowly impairs memory, thinking, and cognitive skills leading to dependency on family or health care services and finally to death.

Suggested research criteria define AD by pathological changes in the brain that exist in the absence of symptoms in as preclinical AD,^2^ or with symptoms as prodromal AD (related to a mild cognitive impairment, MCI)^3^ or as dementia due to AD.^4,5^ Amyloid deposition can be assessed by cerebrospinal fluid (CSF)^6^ and positron emission tomography (PET).^7^ However, there is no simple diagnostic tool to set a definite diagnosis of AD, and there are discussions about whether AD can be defined solely as a biomarker-based entity without assessing symptoms.^8–12^ Furthermore, in contrast to the amyloid cascade hypothesis, more complex AD concepts have been suggested.^13–15^

There are currently two anti-amyloid treatments (AATs) that have shown statistically significant effects on cognition in phase 3 randomized controlled trials, lecanemab and donanemab.^16,17^ Both drugs are antibodies targeting amyloid-β (Aβ) fibril formation and senile plaques. For the moment, lecanemab and donanemab are approved for market access by the Food and Drug Administration in the US. Lecanemab is also approved in Japan, China, Hong Kong, the republic of Korea, United Arab Emirates and Israel. In the UK, the Medicines and Healthcare products Regulatory Agency (MHRA) has granted a marketing authorization for lecanemab and donanemab for the treatment of early AD,^18,19^ while The National Institute for Health and Care Excellence (NICE) has in a preliminary draft stated that the clinical benefits are too small to justify the costs to the National Health Service,^20^ but their final decision is pending (October 2024). The Committee for Medicinal Products for Human Use (CHMP) from the European Medicines Agency (EMA) recommended market access for lecanemab in EU.^21^ For donanemab, the approval process within EMA is ongoing (November 2024). There are discussions about whether the effects are clinically meaningful,^22–24^ and concerns about side effects such as amyloid-related imaging abnormalities (ARIA).^22,25^ Empirical evidence from a randomized study design on their effect beyond the clinical trial follow-up periods of 18 months is lacking^26^ and sustained effect estimates rely on assuming a disease-modifying nature.^27^ Furthermore, there are discussions regarding the pricing of AATs and their budget impact.^26,28^

In recent years, blood-based biomarkers (BBM) of AD^29,30^ have been hypothesized as effective, easily managed and relatively cheap complements to the current more complex and more expensive diagnostic tests as such as CSF and PET. Several studies indicated that BBMs, more specifically plasma p-tau 217, in terms of sensitivity, specificity and predictive values might not only complement, but also potentially replace CSF biomarkers or PET in clinical trials,^31^ or even in clinical practice.^30,32,33^

Systematic reviews on the cost-effectiveness of (diagnostic) interventions for AD^34–37^ have identified several studies that showed the potential for diagnostic interventions to be cost-effective, including a study on different diagnostic strategies to optimize treatment allocation in terms of cost-effectiveness^38^ and a study on CSF prognostic testing and subsequent treatment with cholinesterase inhibitor that was cost-effective.^39^ We identified three additional studies on biomarker-based diagnosis combined with anti-amyloid treatment. Mattke et al. presented a fully hypothetical scenario relying on assumed BBM diagnostic accuracy and assumed AAT effectiveness.^40^ Nguyen et al. showed that BBM with confirmatory PET and lecanemab AAT in PET-positive was not cost-effective compared to the standard of care at its current AAT price of $26,500.^41^ Aye et al. showed that BBM and confirmatory CSF biomarkers to initiate AAT was cost-effective at an AAT price of €5000.^42^

With the recent approval of AAT and real-world data on diagnostic accuracy of BBM in clinical practice there is a need for estimating and replicating the cost-effectiveness of the BBM-AAT pathway in a Scandinavian setting as well as to explore a scenario reflecting BBM diagnostic strategy only without CSF or PET confirmation.

We aim to estimate the potential health-economic impact of amyloid testing and subsequently allocate AAT in individuals with Aβ pathology in persons with MCI due to AD at a memory clinic setting in Scandinavian countries. Since the initiation of AAT is dependent on the result of diagnostic testing for Aβ pathology, the effect of AAT on health and economic outcomes should be included when estimating the impact of these diagnostic tests.^43^ Therefore, we address two research questions: First, is BBM testing cost-effective as a replacement or add-on to CSF compared to the CSF testing, both in combination with AAT? Second, is BBM and/or CSF testing in combination with AAT cost-effective compared to standard of care (SoC) without BBM/CSF testing and without AAT? This study is part of the Precision Medicine in Alzheimer's Disease consortium (PMI-AD) which explores diagnostic pathways at memory clinics for identifying early-stage AD patients.^44^

## Methods

Assessing the cost-effectiveness of a BBM requires evidence of the impact of BBM on the final diagnosis, evidence of the diagnosis on patient management, and evidence of patient management on health-economic outcomes in those with and without the underlying disease.^43^ Crucial is not only the ability to correctly identify people with AD (true positive) and without AD (true negative), but also to avoid incorrect diagnoses of AD (false positive) and missed diagnoses of AD (false negative). In addition, treatment effect evidence is limited to relatively short-term 18-month trial follow-up, relying on assumptions to extrapolate over the relatively long disease course (5–15 years) with a high impact of around the clock care needs in later stages.^45^

In the PMI-AD project's research plan, a cost-effectiveness model based on diagnostic pathways followed by an intervention with AAT was sought.^46^ A health-economic decision model was developed to simulate diagnostic tests for amyloid, natural disease progression and its associated quality of life and care costs of AD. Model input estimates were obtained from various sources, among which was the Dementia Disease Initiation (DDI) a national multi-center Norwegian cohort study, focusing on incipient dementia-related diseases,^47^ which was used to estimate BBM and CSF biomarkers diagnostic accuracy^44,48^ and Gonzalez-Ortiz, Kirsebom et al. (submitted).

We employed a societal perspective by reflecting direct medical sector costs, social care sector costs (including institutional care in nursing homes) and unpaid informal care expressed in monetary terms. Uncertainty was addressed by univariate sensitivity analysis. A lifetime horizon was operationalized by simulating up to the age of 100. Future costs and effects were discounted at an annual rate of 3%.^49^ A willingness-to-pay (WTP) threshold of €94,800 (1,000,000 SEK) was used in the base case, reflecting the level applied in most decisions by the Swedish reimbursement authority The Dental and Pharmaceutical Benefits Agency.^49^ This threshold was assumed also to reflect the minimum savings willing to accept one QALY lost.

The PMI-AD project was ethically approved by Regional Etisk Komite i Norge, reference number 2023/50738.

### Target population and setting

The target population and setting were persons presenting themselves at a memory clinic for diagnosis to determine the cause of their cognitive complaints. Since the recommendation is to start AAT as early as possible, that is MCI due to AD, the base case model assumes that treatment is initiated at the MCI stage in all cases. We target Scandinavian countries Norway and Sweden assuming they are similar in health and social care systems. A starting age of 70 was used, similar to the mean baseline age in the AAT trials.

### Comparators

Three test-treat intervention strategies and one SoC control strategy were addressed. First, CSF Aβ_42/40_ ratio and AAT if Aβ_42/40_ ratio was abnormal. Second, BBM p-tau 217 and AAT in individuals with suspected Aβ pathology. Third, BBM p-tau 217 followed by CSF testing in individuals with suspected Aβ pathology and then AAT if CSF if Aβ_42/40_ ratio was abnormal (i.e., both BBM and CSF biomarkers abnormal). Fourth, SoC reflecting no testing for Aβ pathology and no AAT. For the first research question, the CSF strategy is considered the reference strategy, assuming AAT is already a part of the standard of care. For the second research question, current SoC (without AAT) is considered the reference strategy.

The effect of anti-amyloid treatment was approximated by the 27% difference in disease progression rate expressed as Clinical Dementia Rating-sum of boxes scores between the lecanemab phase 3 intervention and control arm (see Supplemental Table 1)^16^ and the 31% in the donanemab phase 3 trial (Supplemental Table 2).^17^ In the lecanemab trial, treatment took place during MCI due to AD and mild dementia due to AD, while in the donanemab trial there was a pausing/stopping rule based on amyloid clearance. Since the design of the trials differed, but the magnitude of the intervention was rather similar, we therefore in the base case could assume a reduction by 30% in the conversion from MCI-AD to mild-AD and from mild AD to moderate AD. Treatment was also assumed to continue beyond both trials’ follow-up period of 18 months up to moderate dementia, and its effect was assumed to be sustained without any effect waning. No effect was assumed during moderate AD and severe AD. No treatment discontinuation was simulated in the base case. Monitoring during the first year of treatment included physician visits and MRI every 3^rd^ month. Since the side effects of ARIA most often occur in the first year,^16,17^ we assumed an extra physician visit and an extra MRI every subsequent year during treatment.

### Analytics and assumptions

For diagnostic accuracy of CSF and BBM we considered Aβ_42/40_ ratio to be either normal or abnormal (i.e., dichotomous) and it was assessed in terms of sensitivity and specificity, and positive and negative predicted values (PPV and NPV respectively). Estimates were obtained from the DDI cohort study, which included 994 persons across different stages of AD between 40 and 80 years of age, primarily recruited from 5 memory clinics and advertisements in local news media. At baseline the mean age was 64 years (SD 9.4), 55% were female and the mean MMSE was 26.3 (SD 7.5).

The diagnostic accuracy of CSF (MSD Aβ_42/40_ ratio) was assessed using amyloid PET (^18^F-Flutemetamol) as the reference standard in a subsample of 145 persons in the DDI cohort.^50^ The sensitivity and specificity were 93.1% and 96.3% respectively, and the positive (PPVs) and negative predictive values (NPVs) were 93.1% and 96.3% respectively (Supplemental Tables 3 and 4).

The diagnostic accuracy of plasma BBM (p-tau 217) was assessed using CSF Aβ_42/40_ ratio as a reference standard in a different subsample of 290 persons with MCI in the DDI cohort (Gonzalez-Ortiz, Kirsebom et al., submitted). The sensitivity and specificity were 72.1% and 85.3% respectively, and the PPVs and NPVs of 83.5% and 74.8% respectively (Supplemental Tables 5 and 6) (Gonzalez-Ortiz, Kirsebom et al., submitted). The correspondence between BBM and PET was obtained by combining both accuracy estimates. First, the probability of any combination of BBM, CSF, and PET was estimated using the conditional probabilities assuming they are independent (Supplemental Table 7). Based on these probabilities, the probability of the test (combination) under evaluation (CSF, BBM, BBM-CSF) being positive/negative was estimated, and next the probability of the positive/negative test being true/false was estimated (Supplemental Table 8 for CSF, Supplemental Table 9 for BBM, and Supplemental Table 10 for combination BBM-CSF). Based on the probabilities of combined test outcomes, the prevalence of Aβ pathology in our target population was 0.490, which was applied in all four strategies.

We assumed that people with Aβ pathology responded to AAT with an effect magnitude reflected by the efficacy estimate from lecanemab and donanemab trial outcomes, as the presence of Aβ pathology was a key criterion defining the target population in the AAT trials. We assumed that people without Aβ deposition did not respond to AAT and simulated this as non-AD natural disease progression across stages of MCI and mild, moderate and severe dementia. The AAT trials^16,17^ used PET to identify persons eligible for treatment. We assumed that AAT is as effective after a true positive diagnosis regardless of test (BBM, CSF, or PET).

### Rationale and description of model

A decision tree was used to reflect the diagnostic work-up and a Markov cohort model was used to reflect disease progression. This combination has been previously used.^34,38,41,42^ The Markov disease progression model was operationalized by transitions between MCI and mild, moderate and severe dementia specific for AD and non-AD, and transitions from each severity state to death. Transitions between severity states were conditional on survival. The model structure (Supplemental Figures 1 and 2) was based on the framework and inputs from previously published (open source) models.^51–53^ The model was programmed in TreeAge™ with additional analysis in spreadsheets. The cycle length was one year, and a half-cycle correction was applied. Incremental cost-effectiveness ratio (ICER) was calculated as the cost difference (i.e., costs of the intervention strategy minus the costs of the control strategy) divided by the QALY difference (i.e., QALYs in the intervention strategy minus the QALYs in the control strategy). The incremental net health benefit was calculated as the QALY difference minus the ‘cost difference divided by the willingness to pay threshold’.

For all strategies the model started with applying the proportion of persons across the diagnostic categories (true positives, true negatives, false positives, false negatives) as described above. Then, they entered the MCI starting state. Those with correctly identified AD pathology (true positives) had slowed AD disease progression across the stages of AD with the AAT efficacy estimate applied to the transition probabilities of MCI to mild dementia and mild dementia to moderate dementia. Those correctly diagnosed as Aβ-negative (true negatives) or incorrectly diagnosed as Aβ-positive (false positives) had natural non-AD disease progression. Those incorrectly classified as Aβ-negative (false negatives) had natural AD disease progression.

For the SoC strategy, the prevalence of AD/non-AD and its corresponding disease progression was simulated without diagnostic workup and without subsequent AAT.

Dementia disease progression transition probabilities between states (Supplemental Table 1^1^) were derived from predicted values from the coefficients of an earlier reported ordered logit regression (see Supplemental Table 12) fit to data from the SveDem registry.^52,54^ Transitions were conditional on previous severity state and dementia type (AD or non-AD, with the latter operationalized as other dementia). MCI disease progression transition probability to dementia for those with Aβ-positive were obtained from Vos et al., transformed to annual transition probabilities.^55^ MCI disease progression of those without AD was obtained from a review by Bruscoli and Lovestone,^56^ the average annual conversion risk is about 10%, which was also used in a previous model.^52^

**Table 1.** Mean and total per person lifetime effects across the SoC strategy and the diagnostic strategies followed by AAT based on a simulated cohort of 100,000 persons (some deviation due to rounding).

					Difference to SoC		
	SoC	CSF-AAT	BBM-AAT	BBM-CSF-AAT	CSF-AAT	BBM-AAT	BBM-CSF-AAT
PY alive	11.3	11.7	11.6	11.6	0.4	0.3	0.3
PY nondementia	5.3	5.8	5.7	5.6	0.6	0.4	0.4
PY dementia	6.1	5.9	5.9	5.9	−0.2	−0.2	−0.2
PY mild dementia	3.1	3.3	3.2	3.2	0.2	0.1	0.1
PY moderate dementia	2.5	2.2	2.3	2.3	−0.3	−0.2	−0.2
PY severe dementia	0.5	0.4	0.4	0.4	−0.1	−0.1	−0.1
PY dead	18.7	18.3	18.4	18.4	−0.4	−0.3	−0.2
Deaths	99,945	99,942	99,942	99,942	−3	−3	−3
True positive		47,194	34,299	34,031			
True negative		47,484	41,486	48,459			
False positive		3496	9494	2521			
False negative		1826	14,721	14,989			

AAT: anti-amyloid treatment; BBM: blood-based biomarker; CSF: cerebrospinal fluid; PY: person-year; SoC: standard of care.

It was assumed that people with MCI had the same age-specific mortality risk as the general population. For people with dementia, their relative risk of death specific for mild, moderate and severe dementia compared to normal was applied to the general population age-specific mortality rate. The hazard ratio was obtained from a survival regression model fit to data from the SveDem registry compared to very mild dementia, combined with a hazard ratio between persons with very mild dementia and persons without dementia (see Supplemental Table 13 and the supplement to^52^).

### Resource use and costs inputs

Norway and Sweden are similar in health and social care systems, and thus the modelled setting can be regarded as “Scandinavian”. The cost year is 2019, where 1€ corresponded to 10.545 SEK, to 8.955 NOK and to 1120 US$.

In Norway there are no official tariffs or price lists for the health system. To achieve more standard unit costs, we have used a Swedish tariff with information on all used diagnostic tools, based on the price list for the Karolinska University Hospital in Stockholm and adjusted by GDP per person, similar to the method that was used in another PMI-AD paper.^48^ Since there is no price for the BBMs in clinical practice, a price based on expert opinion was used.

The mean cost per person for AD diagnostic testing was €997 and €1364 for the BBM and CSF strategies respectively and €1550 for the combined BBM-CSF strategy. For SoC without BBM or CSF testing it was €714€ (Supplemental Tables 14 and 15 and^48^).

The care system consists of two levels: living at home and institutionalized. When people live at home the following resources are included: Home care, home medical care, day care and informal care. Irrespective of the care level, costs of diagnosis and treatment are presented as part of the medical sector.

The disease severity state-specific costs are based on the distribution of people with AD in the Swedish care system in each state. This approach has been used in previous models,^51,57^ but the input unit costs and the distribution of the simulated cohort in the case system as well as the amounts of informal care are now updated and are based on a recently published cost of illness report,^58^ and publications from the population-based project Swedish national study on aging and care (SNAC).^51,59,60^ The state-specific costs were not adjusted for age assuming it is the severity stage that drives the costs (Supplemental Table 16).

Since the starting age is 70 in the base case, indirect costs in terms of productivity loss of patients with AD are not included.

The price set for lecanemab in the US was $26,500, which we regard as unrealistic for an AAT in Europe given its expected budget impact.^26^ Accordingly applied price level of about €5000 was used in our model's base case.^38,52,61^ Costs of managing the infusions at clinics and monitoring were added to the price of the AAT (Supplemental Table 17).

### Quality of life inputs

Utilities for the different severity states are derived from Ekman et al.^62^ (see Supplemental Table 18).

### Heterogeneity and uncertainty

Treatment may start earlier than at the age of 70, so thus a scenario with treatment starting at 60 was applied which also accounts for the effects on production losses between age 60–65. The treatment effect was assumed to be the same as for 70-year-olds.

Treatment discontinuation was considered and reflected by 10% and 20% discontinuation in year 1 (0% in the base case).

The base case AAT effect by 30% was varied in different ways. In the base case, it was assumed that the effect of the intervention persists. Since we have no data on the long-term effects, we also tested a conservative scenario with treatment effect waning: intervention effects decrease by 10% and 20% per year. We also varied intervention effects by 100% (best case, no conversion to dementia at all), 45% (50% better than base case), and 15% (50% worse than base case) to explore sensitivity to a broader range of intervention effects. We also simulated treatment initiation in mild AD dementia to see if it is more cost-effective to start with a more severe population. We also tested whether it was cost-effective to treat in moderate AD dementia, assuming the same magnitude of the intervention effect.

After 30 years almost everyone in the model had reached the state of death. The uncertainty in health-economic outcomes increases with the length of the model due to relying on assumptions of sustained treatment effect beyond 18-month trial results. To assess whether the cost-effectiveness is different during the model period, we tested scenarios with shorter periods: 10 and 20 years.

Since no AAT has yet been reimbursed in Europe, there is also no price set in Sweden and other EU countries. Thus, we tested the impact of AAT prices of €2500 and €25,000/year (the latter reflecting the price that has been suggested by the company for lecanemab in the US).

We assumed CSF testing is already part of the standard diagnostic workup in MCI, opposite to assuming no CSF is performed for suspected MCI in the SoC strategy in the base case.

We assumed a weekly subcutaneous injection that can be managed at home.^63,64^ Consequently, AAT strategies have no costs for the administration of infusions.

We tested discount rates of 0% and 5% (compared to 3% in the base case).

Having an incorrect diagnosis (false positive or false negative) may generate a disutility related to anxiety or discomfort from knowing it, both in the short- and long-term perspective.^65–68^ We ad hoc test disutilities at −0.05 for (false positives or false negatives) in all strategies.

We tested an alternative source for diagnostic accuracy of BBM, based on Palmqvist et al.'s paper.^69^ Two options for MCI are here tested: one with the ratio of p-tau217 to non–p-tau217 (with NPVs and PPVs of 94.5% and 84.0% respectively), and one option where the p-tau 217 ratio is combined with the Aβ_42/40_ plasma ratio (the amyloid probability score 2 (APS2)) (with NPVs and PPVs of 90.9% and 88.6% respectively). The same method was applied to estimate the probability of any combination of BBM, CSF, and PET as described in the ‘Analytics and assumptions’ section.

We tested the combination of AAT discontinuation by 10% and waning by 10%.

We tested the combination of AAT discontinuation by 10% and waning by 10% for the subcutaneous alternative.

We tested the cost-effectiveness of lecanemab using its specific starting age (71 years), starting proportion in MCI (61.5%) and mild dementia (38.5%), treatment effect in MCI (28%) and mild dementia (27%) and discontinuation rate (6.9%).

We tested the cost-effectiveness of donanemab using its specific starting age (73 years) starting proportion in MCI (17%) and mild dementia (83%), treatment effect in MCI (30%) and mild dementia (33%) and discontinuation rate (13.1%).

### Ethical permission

PMI-AD was approved by the Regional Ethical Committee in Norway, reference number 2023/50738.

## Results

Total lifetime results for each strategy from the health-economic simulation model are presented in Table 1. In SoC strategy people were estimated to spend 5.3 years without dementia. This was 0.6 years longer in the CSF-AAT strategy and 0.4 years longer in the BBM-AAT and BBM-CSF-AAT strategies. Time spent in moderate and severe dementia was shorter in the CSF-AAT, BBM-AAT, and BBM-CSF-AAT strategies compared to the SoC strategy. Life expectancy in CSF-AAT strategy was 0.4 years longer and in BBM-AAT and BBM-CSF-AAT strategies 0.3 years longer compared to the SoC strategy. The proportions of true positives were about 28% lower with the BBM-AAT and BBM-CSF-AAT strategies as compared to the CSF-AAT-strategy. There were small differences between years 20 and 30, but after 10 years, the effects on mortality were rather strong (Supplemental Table 19).

The cost was highest in the CSF-AAT strategy, QALY gains were largest in the CSF-AAT strategy.

For research question 1 (Table 2) compared with CSF-AAT both BBM-AAT and BBM-CSF-AAT strategies resulted in QALYs lost at lower costs, which reflects cheaper but also show worse health outcome than the CSF-AAT strategy. The corresponding ICER for BBM-AAT was 27,494€, which was lower than the threshold of €94,800 minimum cost savings willing to accept one QALY loss (resulting in a negative incremental net health benefit). The corresponding ICER for BBM-CSF-AAT was 109,360€, which was higher than the threshold (resulting in a positive incremental net health benefit). When only diagnostic costs were considered, the cost per true positive case was lowest for the CSF pathway while it for a true negative case was lowest for the BBM pathway.

**Table 2.** Research question 1: mean per person lifetime incremental costs, QALYs, net benefits, and ICER for the diagnostic strategies BBM-AAT and BBMCSF-AAT strategies using CSF-AAT as comparator and diagnostic costs per true positive and true negative case (some deviations due to rounding).

Strategy	Cost (€)	QALY	Net monetary benefit	Net health benefit	Incremental cost vs. CSF-AAT	Incremental QALY vs. CSF-AAT	ICER vs. CSF-AAT	Incremental net health benefit vs. SoC	Diagnostic costs per true positive case of AD	Diagnostic costs per true negative case of AD
CSF-AAT	271,114	6.484	343,540	3.624	n/a	n/a	n/a	n/a	2889	2872
BBM-AAT	268,655	6.394	337,520	3.560	−2459	−0.089	27,494	−0.06	2906	2403
BBM-CSF-AAT	261,130	6.392	344,869	3.638	−9984	−0.091	109,360	0.01	4556	3200

AAT: anti-amyloid therapy; BBM: blood-based biomarker; CSF: cerebrospinal fluid; ICER: incremental cost-effectiveness ratio; QALY: quality-adjusted life year; vs.: versus; n/a: not applicable;

For research question 2 (Table 3) compared with SoC (i.e., no diagnostics and no AAT) all three strategies (CSF-AAT, BBM-AAT, and BBM-CSF-AAT) resulted in QALY gains at higher costs (more expensive but with better health outcomes) with corresponding ICERs being €109,981, €140,992, and €110,222 per QALY gained. These were respectively 16%, 49%, and 16% higher than the maximum willingness to pay for one QALY gained (resulting in a negative incremental net health benefit).

**Table 3.** Research question 2: mean per person lifetime incremental costs, QALYs, net benefits, and ICER for the test-treat strategies using SoC as comparator strategy (some deviations due to rounding).

Strategy	Cost (€)	QALY	Net monetary benefit	Net health benefit	Incremental cost vs. SoC	Incremental QALY SoC	ICER vs. SoC	Incremental net health benefit vs. Soc
SoC	235,114	6.156	348,509	3.676	n/a	n/a	n/a	
CSF-AAT	271,114	6.484	343,540	3.624	36,000	0.327	109,981	−0.05
BBM-AAT	268,655	6.394	337,520	3.560	33,541	0.238	140,992	−0.12
BBM-CSF-AAT	261,130	6.392	344,869	3.638	26,016	0.236	110,222	−0.04

AAT: anti-amyloid therapy; BBM: blood-based biomarker; CSF: cerebrospinal fluid; ICER: incremental cost-effectiveness ratio; QALY: quality-adjusted life year; SoC: standard of care; vs.: versus; n/a: not applicable.

**Figure 1.**
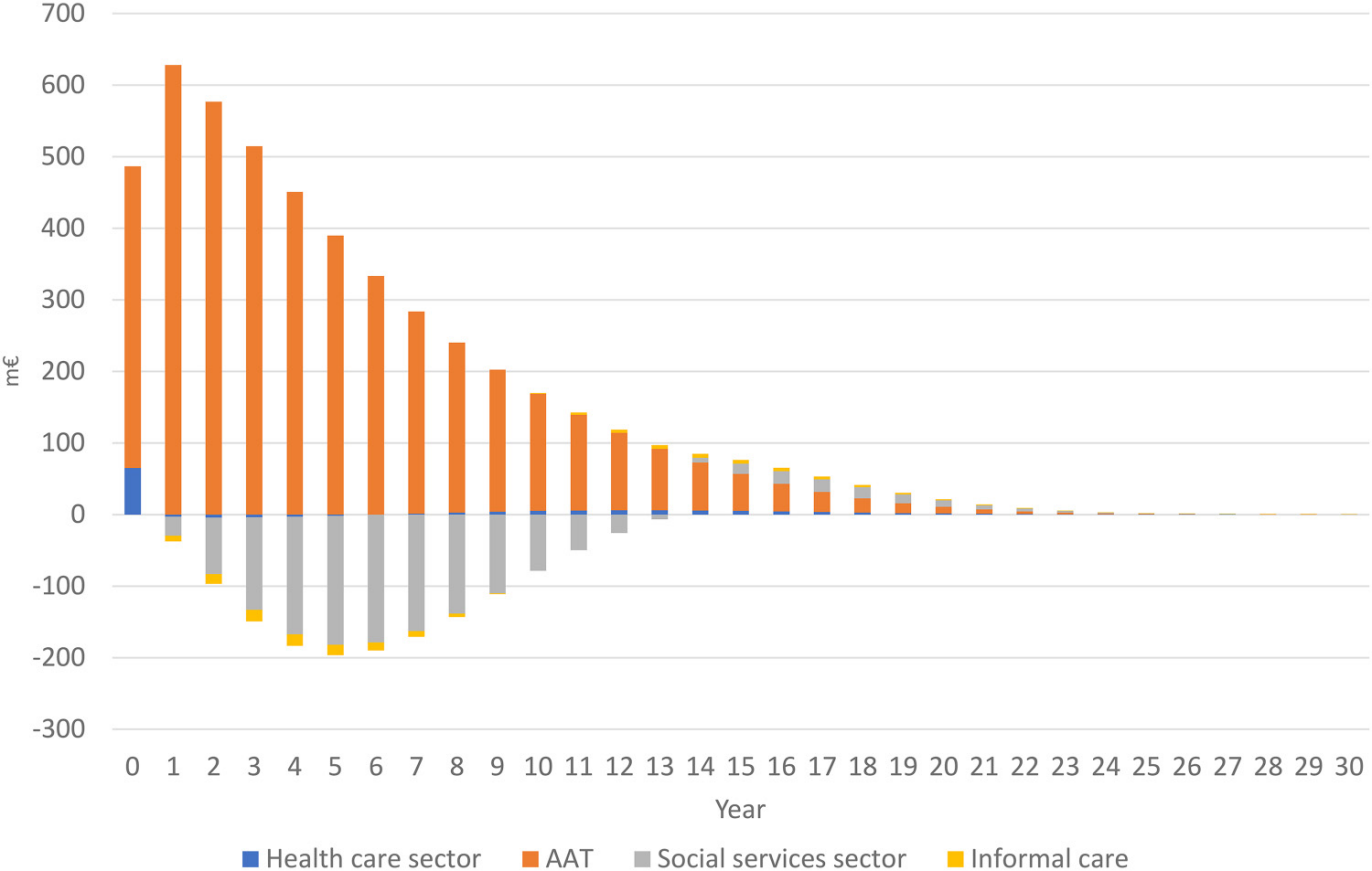
Differences in costs (million €) in a cohort of 100,000 individuals since baseline during the model period with the CSF-AAT strategy versus SoC strategy. Cycles 0 and 30 are half-cycle corrected.

In the SoC strategy, the municipalities had by far the heaviest economic burden of the costs (76%) (Table 4). With the CSF-AAT strategy, the cumulated cohort costs for institutional care decreased by 10% compared to SoC strategy. Without AAT, the costs in the medical sector are low both in absolute and relative terms. Since the medical sector will likely pay for the AAT and their related costs (administration, monitoring), in a cohort of 100,000 individuals the costs in that sector will increase from €2.8 million to €7.6 m (or on average €27,653 to €76,389 per individual) (Table 4). Figure 1 illustrates the incremental costs during the simulated period and the significant impact of the AAT-related costs, particularly during the first years. Since treatment starts in MCI, other care-related costs are low initially.

m€: million €; AAT: anti-amyloid therapy.

**Table 4.** Cumulated lifetime costs (million €) for a cohort of 100,000 (some deviation due to rounding).

Strategy:	SoC		CSF-AAT				BBM-AAT				BBM-CSF-AAT			
	m€	% of total	m€	% of total	Difference vs. SoC	% change	m€	% of total	Difference vs. SoC	% change	m€	% of total	Difference vs. SoC	% change
**Municipalities**														
Institutional care	12,855	55%	11,570	43%	−1285	−10%	11,921	44%	−934	−7%	11,929	46%	−927	−7%
Home care	4955	21%	5017	19%	62	1%	5000	19%	45	1%	5000	19%	45	1%
All municipal costs	17,811	76%	16,587	61%	−1223	−7%	16,922	63%	−889	−5%	16,929	65%	−882	−5%
**Medical sector (regions)**														
Hospital care	422	2%	442	2%	20	5%	436	2%	14	3%	436	2%	14	3%
Open care	1678	7%	1714	6%	36	2%	1704	6%	26	2%	1704	7%	26	2%
Drugs (except AAT)	593	3%	579	2%	−14	−2%	583	2%	−10	−2%	583	2%	−10	−2%
Diagnostics	71	<1%	136	1%	65	91%	100	<1%	28	40%	155	1%	84	117%
AAT costs	0	0%	4767	18%	4767		4222	16%	4222		3438	13%	3438	
**All medical sector costs**	2765	12%	7639	28%	4874	176%	7045	26%	4280	155%	6317	24%	3551	128%
**Informal care**	2936	12%	2885	11%	−50	−2%	2899	11%	−37	−1%	2899	11%	−36	−1%
														
**Total**	23,511	100%	27,111	100%	3600	15%	26,866	100%	3354	14%	26,144	100%	2633	11%

*the cost per case can be calculated by dividing the figures by 100,000

m€: million €; AAT: anti-amyloid therapy; BBM: blood-based biomarker; CSF: cerebrospinal fluid; SoC: standard of care.

The one-way sensitivity analyses (Tables 5 and 6) illustrated that the health-economic outcomes were relatively sensitive to different model inputs and assumptions (variation on AAT price, disutilities, waning and intervention effect). A shift from infusions to subcutaneous injections had a great impact on the ICER, while the effect of discontinuation was relatively low. There were small effects between 20 and 30 years of model length compared to the higher ICER after 10 years. A treatment starting at age 60 seems advantageous, and also to prolong treatment during the moderate dementia state (although there are no efficacy data available for treatment during moderate AD). Even with a complete halt of the conversion from MCI to dementia (100% efficacy), there were no cost savings, mainly because of the imperfect diagnostic accuracy, the treatments costs and care costs in life years saved. The higher cost of diagnostic work-up in SoC had a very small effect. Using an alternative source for BBM diagnostic accuracy^69^ resulted in a somewhat smaller ICER for the BBM-AAT strategy versus the CSF-AAT strategy. The lecanemab and donanemab specific alternatives (with start also in mild AD and discontinuation) resulted in higher ICERs and lower incremental net health benefits (Table 5). Assumptions of disutilities for incorrect diagnosis resulted in a higher ICER for the CSF-AAT (Table 5), BBM-AAT and BBM-CSF-AAT strategies (Table 6).

**Table 5.** Mean per person lifetime outcomes from one-way sensitivity analyses of the CSF-AAT strategy compared to the SoC strategy (WTP = €94,800) (some deviation due to rounding).

Sensitivity analysis	Incremental costs	Incremental QALYs	ICER	Incremental net health benefits vs. SoC
Base case	36,000	0.327	109,981	−0.052
AAT start 60 (70 in base case)	35,426	0.383	92,566	0.009
AAT start at 65(70 in base case)	36,146	0.361	100,058	−0.020
AAT discontinuation 10% (0% in base case)	36,366	0.299	121,583	−0.085
AAT discontinuation 20%	36,735	0.271	135,549	−0.116
AAT waning 10% (0% in base case)	37,087	0.230	160,968	−0.161
AAT waning 20%	37,816	0.176	214,670	−0.223
AAT waning −10%	34,389	0.532	64,679	0.169
AAT waning −20%	32,471	0.830	39,139	0.487
AAT treatment effect 100% (30% in base case)	19,391	2.375	8165	2.170
AAT treatment effect 15%	38,451	0.154	249,277	−0.251
AAT treatment effect 45%	32,764	0.577	56,831	0.231
AAT start in Mild AD-dementia (MCI in base case)	17,999	0.129	139,296	−0.061
AAT also in moderate AD	35,181	0.343	102,523	−0.028
Model length 10 years (30 in base case)	28,714	0.197	145,782	−0.106
Model length 20 years	35,543	0.321	110,836	−0.054
AAT price €2500/year (€5000 in base case)	26,963	0.327	82,375	0.043
AAT price €25,000/year	108,291	0.327	330,835	−0.815
Diagnostic cost for SoC as for CSF	35,351	0.327	107,998	−0.046
AAT by subcutaneous weekly (infusion in base case)	9238	0.327	28,222	0.230
Discount rate 0% (3% in base case)	42,630	0.433	98,358	−0.016
Discount rate 5%	32,703	0.276	118,643	−0.069
Disutilities 0.05 for false positive and false negative cases (0 in base case)	36,000	0.317	113,472	−0.062
AAT discontinuation 10% and AAT waning 10%	36,884	0.242	152,117	−0.147
AAT discontinuation 10% and AAT waning 10% subcutaneous weekly	11,335	0.242	46,748	0.123
Lecanemab trial population and discontinuation inputs	30,392	0.205	148,114	−0.115
Donanemab trial inputs	22,240	0.139	160,408	−0.096

AAT: anti-amyloid therapy; CSF: cerebrospinal fluid; ICER: incremental cost-effectiveness ratio; QALY: quality-adjusted life year; SoC: standard of care.

**Table 6.** One-way sensitivity analyses of BBM-AAT and BBM-CSF-AAT strategies compared to the SoC strategy (WTP = €94,800) (some deviation due to rounding).

Sensitivity analysis	Incremental costs vs. SoC	Incremental QALYs vs. SoC	ICER vs. SoC	Incremental net health benefits vs. SoC
BBM-AAT strategy: base case	33,541	0.238	140,992	−0.116
BBM-AAT strategy: alternative PPVs and NPVs: APS2^69^	37,537	0.310	120,548	−0.084
BBM-AAT strategy: alternative PPVs and NPVs: p-Tau 217-ratio^69^	42,199	0.324	130,250	−0.121
BBM-AAT strategy versus SoC strategy: disutilities 0.05 for false positive and false negative cases	33,541	0.156	215,312	−0.198
BBM-CSF-AAT strategy: base case	26,016	0.236	110,222	−0.038
BBM-CSF-AAT strategy: alternative PPVs and NPVs: APS2^69^	31,423	0.308	101,889	−0.023
BBM-AAT strategy: alternative PPVs and NPVs: p-Tau 217-ratio^69^	33,645	0.298	112,984	−0.057
BBM-CSF-AAT strategy: Disutilities 0.05 for false positive and false negative cases	26,016	0.182	142,958	−0.092

AAT: anti-amyloid therapy; BBM: blood-based biomarker; CSF: cerebrospinal fluid; ICER: incremental cost-effectiveness ratio; QALY: quality-adjusted life year; SoC: standard of care; PPV: positive predictive value; NPV: negative predictive value.

A WTP level of €200,000 per gained QALY may in Sweden be accepted for rare disorders (which is not the case for AD), but even in this case, the threshold level did not reach the suggested price level for lecanemab in the US,^70^ irrespective if it is the base case (infusion) or the weekly subcutaneous injections at home (Table 7). Our selected price level in the base case (€5000) seems reasonable regarding the Swedish WTP level.

**Table 7.** AAT price threshold (€) at which the incremental cost-effectiveness ratio reaches the WTP-threshold (€).

WTP-level	AAT price threshold: base case (infusion)	AAT price threshold: weekly subcutaneous
0 (cost saving)	n/a	2444
25,000	n/a	4708
50,000	n/a	6972
75,000	1832	9236
94,800*	3625	11,029
100,000	4096	11,500
125,000	6360	13,764
150,000	8624	16,028
200,000	13,152	20,556
300,000	22,208	29,612

*assumed Swedish WTP-level

WTP: willingness to pay; AAT: anti-amyloid therapy.

## Discussion

In this study we analyzed the potential cost-effectiveness of treatment (AAT) integrated into different diagnostic pathways. The BBM-CSF-AAT test-treat pathway seems to be a potentially cost-effective strategy if AAT was assumed part of the SoC. Compared to the current SoC, and with the assumed price of AAT (€5000/year), the potential cost-effectiveness of two diagnostic strategies (CSF Aβ positivity followed by AAT and BBM-CSF combination followed by AAT) was 16% higher than the assumed maximum WTP threshold in Sweden.

Diagnostic accuracy of BBM is often obtained in research settings with participants recruited using narrow inclusion criteria in relatively highly controlled research settings. Our diagnostic estimates were obtained from local memory clinics and academic settings. The cost-effectiveness of the scenario with a BBM as a screening step before CSF testing seems advantageous but it needs more confirmation in real-world settings. Since predictive values of diagnostic tests are driven by disease prevalence, there is a risk of greater numbers of particularly false positives in a primary care environment with lower prevalence. The cost-effectiveness could be further influenced by choosing different cut-offs at which BBM amyloid level is considered abnormal or if persons experience a disutility due to false diagnosis, as has been explored in previous studies on CSF.^38,68^

The cost-effectiveness estimates were driven both by the efficiency of the treatment and the accuracy of the diagnostic test. Diagnostic accuracy translated to a distribution across true positive, true negative, false positive (overtreatment) and false negative (undertreatment). The balance between over- and undertreatment drives the cost-effectiveness, with the former resulting in unnecessary treatment side effects and costs and the latter missing out on the opportunity to gain QALYs from the treatment effect. The CSF-AAT and BBM-CSF-AAT strategies versus SoC strategy have very similar ICERs. The BBM-CSF-AAT strategy had lower costs (CSF confirmation prevented false positives related to BBM and corresponding unnecessary treatment costs). However, it had less gained QALYs than the CSF-AAT strategy (CSF prevented both false positive and false negative cases related to BBM).

If treatment would be cheap and highly effective, maximizing a positive diagnosis (i.e., a ‘liberal’ diagnostic approach) would improve cost-effectiveness as the opportunity loss (i.e., missing out the benefits of treatment in case of true positive) is higher than the cost of unnecessary treatment of false positives. Vice versa, if treatment is expensive (like the US price for lecanemab) and its effect is modest (as with both lecanemab and donanemab), maximizing a negative diagnosis (i.e., a ‘conservative’ diagnostic approach) would improve cost-effectiveness as it prevents expensive unnecessary treatment in true negatives at a loss opportunity for a small treatment gain (false negatives).

Since AAT claims to impact the underlying course of AD (“disease modifying therapy”) there are hopes that AATs have the potential to result in cost-savings from a societal viewpoint. However, this was not the case in any of the sensitivity analysis scenarios (not even with a 100% treatment effect), which aligns with other health-economic evaluations.^42,51,52,61,71,72^ However, given the WTP level and the assumed treatment price somewhat lower than in our assumed price, AAT could be considered cost-effective. Nevertheless, our assumed AAT price is already much lower than the presented prices for lecanemab in the US and Japan (26,500 US$ and 20,000 US$ respectively^70,73^).

Empirical long-term data on mortality from the lecanemab and donanemab studies are lacking. Assumptions of mortality have been shown to heavily impact the cost-effectiveness of AD treatment to delay disease progression.^51^ The sensitivity analysis illustrated assumptions about waning, AAT costs (treatment administration method infusion versus subcutaneous administration) and AAT effects had a strong impact on the cost-effectiveness outcomes, stressing transparency in testing and reporting them. The sensitivity of cost-effectiveness estimates to these assumptions with limited or no empirical evidence questions the results of modeling studies. However, the relatively long natural disease period of AD (about 5–15 years) is hard to cover by the follow-up period of a randomized controlled trial (currently 18 months).^74^ Initiatives have been undertaken to explore differences in AD modelling^75^ and develop open-source models for AD^53,61^ to support the transparency and credibility of models in AD.

The higher ICER of donanemab compared to lecanemab seems driven by that the donanemab trial had treatment initiated at a somewhat older age, a larger proportion of the starting population had mild dementia and a larger discontinuation. However, donanemab had a somewhat better treatment effect (better net health benefits than for lecanemab). We note we did not detail the treatment discontinuation due to amyloid clearance for donanemab, limiting the comparison of the health-economic outcomes between both drugs.

Our results align with previous modeling studies that assessed the cost-effectiveness of BBM combined with AAT.^41,42^ Compared to Nguyen et al.^41^ we also found the strategy of CSF testing and AAT treatment not cost-effective when using a similar treatment price, and cost-effective if the treatment price was significantly lower. The price threshold at which CSF-AAT was cost-effective differed due to differences in assumptions on sustained treatment effect and the US compared to Swedish health-economic inputs and willingness to pay threshold. Compared to Aye et al.^42^ we also found a minor cost difference between CSF-AAT and BBM-CSF-AAT diagnostic strategies. However, our study showed a larger QALY difference of 0.091 (compared to −0.002 in Aye et al.), which could be explained by the larger change from true positive to false negative and its consequential missed QALY benefits due to undertreatment. Like our study they created the necessary condition of a lower price for AAT for diagnostic strategies to become potentially cost-effective.

Based on the age-specific prevalence of Aβ pathology^76^ more than 200,000 persons in Sweden are expected to have MCI due to AD and another 45,000 have mild AD dementia. Although a smaller proportion is considered eligible for treatment (e.g., exclusion by co-morbidity), the target population size (assumed 100,000 in our study) can still be unrealistic regarding budget requirements.^26^ Estimates from the US indicated that 17% could be eligible for AAT.^77^ Corresponding estimates from Sweden^78^ are 27%. However, these figures are from highly specialized centers and the proportion that is eligible for treatment at the general population level is still unknown since the focus in clinical work so far has been to identify people with dementia, not MCI. Being an *APOE* ɛ4 carrier increases the risk for ARIA. The way of managing this risk will influence the size of the target population for AAT. One scenario may be that *APOE* ɛ4 homozygotes will be excluded from AAT. Although the prevalence of homozygotes in MCI^79,80^ is low, the target population for treatment could in this case be reduced by approximately 5–10%.

Availability of AAT might trigger additional people with subjective memory concerns to seek medical advice compared to the current situation without available AAT, increasing the demand for diagnostic work-up in primary care, which already works under stressed conditions.^81^ This likely results in a simple referral process such as testing for cognitive impairment, exclusion of AAT contraindications (anticoagulant therapy, cerebrovascular disease verified by CT scan or MRI and severe comorbidities) and a positive BBM^30^ assuming validated for clinical practice.^69^ Such ‘liberal’ diagnostic process in primary care will forward the diagnostic burden to memory clinics. It has been shown, that the capacity of memory clinics in Sweden and other countries is limited,^82^ creating a hurdle for access to AAT, which requires additional resources for administration and safety monitoring. New technological devices and web-based applications to support the diagnostic process are under development, but their effectiveness and cost-effectiveness in clinical practice are still unknown.^83^

Currently, AAT is administrated as intravenous infusions every 2^nd^ or 4^th^ week. There will also be regulations for treatment monitoring with frequent visits and repeated MRI, which may influence the endurance among patients and caregivers, and by that, compliance and cost-effectiveness will be difficult to predict. There are studies supporting that AAT may be available as subcutaneous injections once a week.^63^ Besides reducing the burden on memory clinics, it will potentially, as shown in the sensitivity analysis, also have a strong impact on the cost-effectiveness.

In Sweden, due to the care budget being organized in silos (such as the medical care sector and social care sector) the social care sector would benefit from AAT as a reduction in institutional long-term care could be expected. The medical care sector, who so far have relatively low costs related to AD will carry the burden for AAT treatment costs. Even if reimbursement authorities accept AAT, payers will likely experience difficulties with managing their budget. This requires discussions about budget impact and budget silo limitations. Alternative paying models have been suggested, where economic risk is spread over time and between payers and AAT producers.^84^

### Strengths and limitations

The strength of our model is the use of empirical data on diagnostic evidence from memory clinics all over Norway and not only highly specialized research centers, giving better opportunities to reflect “the real world” at memory clinics. The initial phase in the diagnostic process (BBM testing in addition to some cognitive testing as well as MRI) for the two BBM-AAT strategies could also take place in primary care. However, the diagnostic accuracy (PPV and NPV) in our model like in many other studies, is based on data obtained in a memory clinic setting. In primary care, the prevalence of cognitive impairment is lower than in memory clinics, which will result in a lower PPV (but a higher NPV).

The validity of BBMs is under evaluation. Concordance between BBM p-tau 181 and CSF Aβ_42/40_ ratio was found to be limited (50%) in a memory clinic setting,^85^ questioning its validity to detect early Aβ pathology.^29^ However, BBM p-tau 217, as in our study, seems to be a better biomarker of early AD pathology.^32,69^ Our cost-effectiveness estimates rely on the assumption that AAT is equally effective regardless of the strategy used to identify eligible patients (i.e., BBM-, CSF-, or PET-based approach), which might not be the case due to differences in the temporal evolution of these biomarkers with underlying disease pathology).

Model-based economic evaluation typically include several assumptions, uncertainties and simplifications of the “real-world”.^86^ The study populations in clinical trials are selected as it is necessary to identify a target population for a specific treatment. However, when treatment is taking place in clinical practice, there may be differences between the populations, making translations to clinical practice problematic. The life-long extrapolation of short-term efficacy evidence is in any model a challenge. Registry follow-up studies and pragmatic trials are expected to generate empirical evidence to provide some support for assumptions on sustained treatment effects and side effects beyond trial follow-up periods, as well as effectiveness in real-world clinical practice, although they also rely on assumptions if not randomized. As in most economic models, we are combining inputs from different sources which may impact the outcomes of our model.

Although our model assumes that removing amyloid from the brain has a positive effect on AD progression, it does not imply that we regard the amyloid cascade hypothesis as the only cause of AD. The great effect of amyloid removal in the trials combined with the rather modest effects on disease progression^87^ may indicate that AD is a complex diagnostic entity. It does imply our analysis assumes the effects observed in the lecanemab and donanemab trials are unbiased and can be extrapolated beyond the trial follow-up period, although there have been concerns of functional unblinding,^87^ biased observation of mortality^88^ and conflict of interest in judging the clinical value of these treatments.^88^

In our model we have costs for monitoring treatment (to among other things detect side-effects like ARIAs), but we have not included cost for treatment of side-effects, since we have no data on that. Our treatment cost may therefore be underestimated.

Furthermore, since long-term data are lacking in trials and naturally also in clinical practice (the use in the US is still on a low and short-term level), it is not easy to estimate future costs of managing treatment effects and risks of AAT. Even if PET would be desirable for relating effects on brain amyloid versus cognition and function, it is questionable, given PET´s availability (and costs) whether this will be the case. For ARIA detection, the frequency of MR scans is also unknown, even if it is realistic to assume that the number of follow-ups after 1–2 years can be reduced, since such effects come early.

In the lecanemab and donanemab trials, there were different stopping rules. In the lecanemab trial, treatment took place during MCI due to AD and mild dementia due to AD (and consequently should stop when reaching moderate dementia due to AD, while for donanemab, treatment stopped (or rather paused) when amyloid clearance was achieved. Our model cannot discriminate between these design differences which is a limitation.

Institutionalization in our model was only linked to AD, but people with AD might have been admitted for other reasons such as frailty and comorbidities. Therefore, some of the institutionalization prevented by our model might not be causally related to AAT, implying the benefits of AAT are possibly overestimated.

Our target population (MCI and abnormal amyloid) differed from the lecanemab and donanemab trials, which had both Aβ-positive MCI and mild dementia. This overestimated the cost-effectiveness as our sensitivity analysis showed a higher ICER when starting AAT in mild AD dementia. Furthermore, discontinuation was not the case in our base case, but it was tested in the sensitivity analysis at two levels (10 and 20%) to better reflect the situation in the lecanemab and donanemab trials.

### Conclusions

The BBM-CSF-AAT diagnostic test-treat strategy is potentially cost-effective when assuming AAT is part of SoC and priced at €5000 per individual per year (which is lower than the prices suggested by the pharmaceutical companies). Both the CSF-AAT strategy and BBM followed by confirmatory CSF testing and AAT were potentially not cost-effective as they exceeded the assumed maximum willingness to pay threshold with 16%. Discussions on the budget impact on different payers are needed to manage an introduction of AAT.

## Supplemental Material

sj-docx-1-alz-10.1177_13872877251323231 - Supplemental material for Cost-effectiveness of diagnosing and treating patients with early Alzheimer's disease with anti-amyloid treatment in a clinical setting Supplemental material, sj-docx-1-alz-10.1177_13872877251323231 for Cost-effectiveness of diagnosing and treating patients with early Alzheimer's disease with anti-amyloid treatment in a clinical setting by Anders Wimo, Ron Handels, Kaj Blennow, Bjørn-Eivind Kirsebom, Per Selnes, Jaka Bon, Andreja Emersic, Fernando Gonzalez-Ortiz, Milica Gregoric Kramberger, Anders Sköldunger, Andreja Speh, Santiago Timón-Reina, Ellen Vromen, Pieter Jelle Visser, Bengt Winblad and Tormod Fladby in Journal of Alzheimer's Disease
