# Supplementary material for: Cost-effectiveness of diagnosing and treating patients with early Alzheimer's disease with anti-amyloid treatment in a clinical setting
Source: J Alzheimers Dis. 2025 Mar 20;104(4):1167–84. doi: 10.1177/13872877251323231 (PMC12231775; doi:10.1177/13872877251323231)
Supplement: sj-docx-1-alz-10.1177_13872877251323231 - Supplemental material for Cost-effectiveness of diagnosing and treating patients with early Alzheimer's disease with anti-amyloid treatment in a clinical setting [file sj-docx-1-alz-10.1177_13872877251323231.docx]

**Supplemental Material**

**Cost-effectiveness of diagnosing and treating patients with early Alzheimer’s disease with anti-amyloid treatment in a clinical setting**

**Supplemental Table 1.** Intervention effect by lecanemab.

|  | **CDR-SOB baseline (range 0-18)** | **CDR-SOB 18 months***  **(range 0-18)** | **Difference change from baseline** | **Relative change** | **Relative change %** |
| --- | --- | --- | --- | --- | --- |
| **Lecanemab** | 3.17 | 4.38 | 1.21 |  |  |
| **Placebo** | 3.22 | 4.88 | 1.66 |  |  |
| **Diff** | 0.05 | 0.5 | 0.45 | 0.45/1.66 | 27% |

*Derived from Mean change and baseline CDR-SOB

**Supplemental Table 2.** Intervention effect by donanemab.

|  | **CDR-SOB baseline**  **(range 0-18)** | **CDR-SOB 18 months* (range 0-18)** | **Difference change from baseline** | **Relative change** | **Relative change %** |
| --- | --- | --- | --- | --- | --- |
| **Donanemab** | 3.92 | 5.25 | 1.33 |  |  |
| **Placebo** | 3.89 | 5.80 | 1.91 |  |  |
| **Diff** | 0.03 | 0.55 | 0.58 | 0.58/1.91 | 31% |

*Derived from Mean change and baseline CDR-SOB

**Supplemental Table 3.** Diagnostic table for dichotomized BBM and CSF amyloid level being abnormal (positive) and normal (negative) levels. Chi-square test p<0.001.

|  | Amyloid PET (^18^F-Flutemetamol) negative | Amyloid PET  (^18^F-Flutemetamol) PET positive | All |
| --- | --- | --- | --- |
| CSF Aβ_42/40_ negative | 52 (TN) | 2 (FN) | 54 |
| CSF Aβ_42/40_ positive | 2 (FP) | 27 (TP) | 29 |
| All | 54 | 29 | 83 |

**Supplemental Table 4.** Sensitivity, specificity and predictive values of CSF using PET as reference standard.

| **Sensitivity** | 93.1% |
| --- | --- |
| **Specificity** | 96.3% |
| **PPV** | 93.1% |
| **NPV** | 96.3% |

**Supplemental Table 5.** Diagnostic table for dichotomized BBM and CSF amyloid level being abnormal (positive) and normal (negative) levels, chi-square test p<0.001 (Gonzalez-Ortiz, Kirsebom et al, submitted).

|  | CSF Aβ_42/40_ negative | CSF Aβ_42/40_ positive | All |
| --- | --- | --- | --- |
| BBM p-tau 217 negative | 122 (TN) | 41 (FN) | 163 |
| BBM p-tau 217 positive | 21 (FP) | 106 (TP) | 127 |
| All | 143 | 147 | 290 |

**Supplemental Table 6.** Sensitivity, specificity and predictive values of BBM using CSF as reference standard (Gonzalez-Ortiz, Kirsebom et al, submitted).

| Sensitivity | 72.1% |
| --- | --- |
| Specificity | 85.3% |
| PPV | 83.5% |
| NPV | 74.8% |

**Supplemental Table 7.** Probability of any combination of BBM, CSF and PET based on diagnostic evidence from BBM-CSF and from CSF-PET assuming they are independent.

| **BBM** | **Value^1^** | **Estimate^*^** | **CSF** | **Value^**^** | **Estimate^**^** | **PET** | **Value^***^** | **Estimate^***^** | **Combination^****^** |
| --- | --- | --- | --- | --- | --- | --- | --- | --- | --- |
| + | 0.438 | Prop. pos. | + | 0.835 | PPV | + | 0.931 | PPV | 0.340 |
| + | 0.438 | Prop. pos. | + | 0.835 | PPV | - | 0.069 | 1-PPV | 0.025 |
| + | 0.438 | Prop. pos. | - | 0.165 | 1-PPV | + | 0.037 | 1-NPV | 0.003 |
| + | 0.438 | Prop. pos. | - | 0.165 | 1-PPV | - | 0.963 | NPV | 0.070 |
| - | 0.562 | Prop. neg. | + | 0.252 | 1-NPV | + | 0.931 | PPV | 0.132 |
| - | 0.562 | Prop. neg. | + | 0.252 | 1-NPV | - | 0.069 | 1-PPV | 0.010 |
| - | 0.562 | Prop. neg. | - | 0.748 | NPV | + | 0.037 | 1-NPV | 0.016 |
| - | 0.562 | Prop. neg. | - | 0.748 | NPV | - | 0.963 | NPV | 0.405 |

^*^ Obtained from BBM-CSF diagnostic accuracy study; Prop. pos. = proportion positive (TP + FP / total);

^**^Prop. neg. = 1 – proportion positive.

^***^ Obtained from CSF-PET diagnostic accuracy study.

^****^ Assuming independence between BBM-CSF and CSF-PET.

‘+’: abnormal amyloid; ‘-’: normal amyloid; BBM: blood-based marker; CSF: cerebrospinal fluid; NPV: negative predictive value; PET: positive emission tomography; PPV: positive predictive value, Prop. pos.: proportion positive; Prop. neg.: proportion negative.

**Supplemental Table 8.** Conditional probabilities for each step in the diagnostic tree for the CSF-AAT strategy, derived from the probability of each combination as presented in Supplemental Table 7.

| CSF | probability | PET | probability (conditional on previous step) |
| --- | --- | --- | --- |
| + | 0.507^*^ | + | 0.931 |
|  |  | - | 0.069 |
| - | 0.493 | + | 0.037 |
|  |  | - | 0.963 |

^*^ Cumulative probability all combinations in which CSF is positive from Supplemental Table 7 (0.340 + 0.025 + 0.132 + 0.010).

‘+’: abnormal amyloid; ‘-’: normal amyloid; CSF: cerebrospinal fluid; PET: positive emission tomography.

**Supplemental Table 9.** Conditional probabilities for each step in the diagnostic tree for the BBM-AAT strategy, derived from the probability of each combination as presented in Supplemental Table 7.

| BBM | probability | PET | probability (conditional on previous step) |
| --- | --- | --- | --- |
| + | 0.438 | + | 0.783^*^ |
|  |  | - | 0.217 |
| - | 0.562 | + | 0.262 |
|  |  | - | 0.738 |

^*^ Cumulative probability all combinations in which BBM is positive and PET is positive (0.340 + 0.003) divided by cumulative probability all combinations in which BBM is positive (0.340 + 0.025 + 0.003 + 0.070) from Supplemental Table 7.

‘+’ = abnormal amyloid; ‘-’: normal amyloid; BBM: blood-based marker; PET: positive emission tomography.

**Supplemental Table 10.** Conditional probabilities for each step in the diagnostic tree for the BBM-CSF-AAT strategy, derived from the probability of each combination as presented in Supplemental Table 7.

| BBM | probability | CSF | probability (conditional on previous step) | PET | probability (conditional on previous step) |
| --- | --- | --- | --- | --- | --- |
| + | 0.438 | + | 0.835^*^ | + | 0.931** |
|  |  |  |  | - | 0.069 |
|  |  | - | 0.165 | + | 0.037 |
|  |  |  |  | - | 0.963 |
| - | 0.562 | n/a |  | + | 0.262 |
| - |  | n/a |  | - | 0.738 |

*Cumulative probability all combinations in which BBM is positive and CSF is positive (0.340 + 0.025) divided by cumulative probability all combinations in which BBM is positive (0.340 + 0.025 + 0.003 + 0.070) from Supplemental Table 7.

^**^ Cumulative probability all combinations in which BBM is positive and CSF is positive and PET is positive (0.340) divided by cumulative probability all combinations in which BBM is positive and CSF is positive and (0.340 + 0.025) from Supplemental Table 7. As the probabilities in Supplemental Table 7 are based on independence between BBM-CSF and CSF-PET, this estimates is the same as the positive predicted value of CSF using PET as reference standard.

‘+’: abnormal amyloid; ‘-’: normal amyloid; BBM: blood-based marker; CSF: cerebrospinal fluid; n/a: not applicable; PET: positive emission tomography.

**Supplemental Table 11.** Predicted transition probabilities for AD and non-AD states based on ^1, 2^ (for transition from MCI to mild dementia) and the ordered probit model coefficients provided in Supplemental Table 1 (for transitions among dementia severity states).

|  |  | **to:** |  |  |  |
| --- | --- | --- | --- | --- | --- |
| AD | States | MCI | Mild | Moderate | Severe |
| from: | MCI | 0.752 | 0.248 | 0 | 0 |
|  | Mild | 0 | 0.706 | 0.293 | 0.001 |
|  | Moderate | 0 | 0.087 | 0.804 | 0.109 |
|  | Severe | 0 | 0 | 0.196 | 0.804 |
|  |  |  |  |  |  |
|  |  |  |  |  |  |
| Non AD |  | to: |  |  |  |
|  | States | MCI | Mild | Moderate | Severe |
| from: | MCI | 0.898 | 0.102 | 0 | 0 |
|  | Mild | 0 | 0.745 | 0.255 | 0.001 |
|  | Moderate | 0 | 0.107 | 0.804 | 0.089 |
|  | Severe | 0 | 0 | 0.230 | 0.770 |

**Supplemental Table 12.** Results of the ordered probit analysis in SveDem (dependent: categorized MMSE mild, moderate, severe; based on 23,146 observations from 13,445 individuals). Copied from. ^2^

| **Parameter** | **Coefficient** | **95% confidence interval** | **p** |
| --- | --- | --- | --- |
| MMSE categorized |  |  |  |
| Mild (MMSE 21-30) | reference |  |  |
| Moderate (MMSE 10-20) | 1.8984 | 1.8479 to 1.949 | <0.001 |
| Severe (MMSE 0-9) | 3.9837 | 3.7343 to 4.233 | <0.001 |
| Diagnosis |  |  |  |
| Alzheimer’s disease | reference |  |  |
| Other dementia | -0.116 | -0.179 to -0.054 | <0.001 |
| Unspecified | -0.025 | -0.086 to 0.036 | 0.418 |
| Cut 1 | 0.542 | 0.510 to 0.574 |  |
| Cut 2 | 3.129 | 3.052 to 3.206 |  |

A selection was made on available transitions (i.e., requiring 2 inter- or extrapolated observations (extrapolation maximum of 3 months into the future). The SveDem obtained 53,880 baseline assessments and 37,491 follow-up assessments of which 25,436 had an MMSE available (68%) (see Handels et al.^3^)

**Supplemental Table 13**. Results of the survival analysis in SveDem registry (52,969 subjects*; 4674 deaths; Weibull distribution) copied from.^2^

| **Parameter** | **Coefficient** | **95% confidence interval** | **p** |
| --- | --- | --- | --- |
| MMSE categorized |  |  |  |
| Very mild (MMSE 27-30) | reference |  |  |
| Mild (MMSE 21-26) | 1.318 | 1.153 to 1.507 | <0.001 |
| Moderate (MMSE 10-20) | 2.419 | 2.122 to 2.757 | <0.001 |
| Severe (MMSE 0-9) | 4.267 | 3.610 to 5.043 | <0.001 |
| Diagnosis |  |  |  |
| Alzheimer’s disease | reference |  |  |
| Other dementia | 1.775 | 1.657 to 1.902 | <0.001 |
| Unspecified | 1.200 | 1.119 to 1.286 | <0.001 |
| Constant | 0.000 | 0.000 to 0.000 | <0.001 |
| Weibull shape parameter p | 7.546 | 7.166 to 7.946 |  |

* This analysis was based on the subsample of persons 60 years or older (n=53,018). A small proportion of this sub-population (<0.1%) had missing MMSE and could not be used for the analysis. Note the difference from the sample used for the ordered probit, which required at least 2 inter- or extrapolated MMSE observations.

**Supplemental Table 14.** Unit costs for the used diagnostic tools at memory clinics in Norway (€2019, GDP per capita adjusted).

|  | Unit cost € | Unit cost SEK |
| --- | --- | --- |
| Clinical examination | 100.96 | 1,064.62 |
| Cognitive screening | 84.69 | 893.06 |
| APOE | 94.43 | 995.76 |
| MRI | 528.75 | 5,575.67 |
| CSF | 554.69 | 5,849.21 |
| PET | 1,149.09 | 12,117.15 |
| BBM* | 186.93 | 1,971.18 |

*expert estimate

Details of the diagnostic costs are published elsewhere.^4^ There are no official tariffs or price lists in the Norwegian health system. Norway and Sweden have rather similar health care systems and we assumed the Swedish tariff, based on the unit price list for the Karolinska University Hospital in Stockholm, is generalizable to Norway after adjusted the cost figures by Gross Domestic Product, GDP) person between Sweden and Norway to get uniform costs. Since there is no set price for the BBMs an estimated price was used, which is similar to the assumed BBM-price in Aye et al.’s paper (200€).^5^ The cost year is 2019, where 1€ corresponded to 10.545 SEK. The corresponding unit costs for the diagnostic tools are seen in Supplemental Table 14.

**Supplemental Table 15.** Costs (€2019) of the diagnostic pathways.

| Pathway | Mean cost € | Diagnostic tests |
| --- | --- | --- |
| CSF | 1363.52 | Clin exam+CS+MRI+CSF+APOE |
| BBM | 996.76 | Clin exam+CS+MRI+BBM+APOE |
| BBM-CSF (for BBM positive) | 1550.45 | Clin exam+CS+MRI+BBM+CSF+APOE |
| SoC | 714.40 | Clin exam+CS+MRI |

Besides the costs that are specifically targeted for AD (CSF and BBM), the diagnostic packages also include costs for a clinical examination, cognitive testing, MRI and APOE. APOE will probably be required due to the ARIA-risks and MRI for the exclusion of cerebrovascular conditions or other conditions that exclude patients from treatment.

**Supplemental Table 16.** Annual costs (€ 2019) per stage.

| Stage | Cost per year (and cycle) |
| --- | --- |
| MCI AD | 10,229 |
| Mild AD | 22,604 |
| Moderate AD | 51,606 |
| Severe AD | 90,196 |

**Supplemental Table 17.** Annual costs related to treatment (€ 2019).

| Stage | Cost | Source |
| --- | --- | --- |
| AAT | 5,000 | Assumed |
| Monitoring year 1 | 2,377 | MRIs + physician visits |
| Monitoring follow-up | 526 | MRI + physiscian visit |
| Managing infusions | 7,404 | Diagnose related group (DRG) (immunotherapy for AD, Karolinska Institutet) |

**Supplemental Table 18.** Utilities per stage.^6^

| Stage | Utility |
| --- | --- |
| MCI AD | 0.82 |
| Mild AD | 0.62 |
| Moderate AD | 0.40 |
| Severe AD | 0.25 |

**Supplemental Table 19.** Cohort mean and total (out of 100,000) lifetime effects across the SoC strategy and the diagnostic strategies followed by AAT after 10-20-30 years.

|  | **30 years** | | | **20 years** | | | | **10 years** | | |
| --- | --- | --- | --- | --- | --- | --- | --- | --- | --- | --- |
| **CSF** | CSF | SoC | Diff | CSF | SoC | Diff | CSF | | SoC | Diff |
| PY alive | 11.7 | 11.3 | 0.38 | 11.5 | 11.2 | -0.36 | 8.6 | | 8.5 | -0.11 |
| PY nondementia | 65.8 | 5.2 | 0.61 | 5.7 | 5.1 | -0.60 | 4.9 | | 4.4 | -0.47 |
| PY dementia | 5.9 | 6.1 | -0.23 | 5.8 | 6.0 | 0.24 | 3.7 | | 4.0 | 0.36 |
| PY mild dementia | 3.3 | 3.1 | 0.21 | 3.3 | 3.1 | -0.20 | 2.3 | | 2.3 | -0.01 |
| PY moderate dementia | 2.2 | 2.5 | -0.35 | 2.2 | 2.5 | 0.35 | 1.2 | | 1.5 | 0.31 |
| PY severe dementia | 0.4 | 0.5 | -0.09 | 0.4 | 0.5 | 0.09 | 0.2 | | 0.2 | 0.06 |
| PY dead | 18.3 | 18.7 | -0.38 | 8.5 | 8.8 | 0.36 | 1.4 | | 1.5 | 0.11 |
| Deaths | 99,942 | 99,947 | -4 | 93,793 | 94,710 | -917 | 38,440 | | 41,359 | -2,920 |
|  |  |  |  |  |  |  |  | |  |  |
| **BBM** | BBM | SoC | Diff | BBM | SoC | Diff | BBM | | SoC | Diff |
| PY alive | 11.6 | 11.3 | 0.27 | 11.4 | 11.2 | -0.26 | 8.5 | | 8.5 | -0.08 |
| PY nondementia | 5.6 | 5.2 | 0.44 | 5.6 | 5.1 | -0.43 | 4.8 | | 4.4 | -0.34 |
| PY dementia | 5.9 | 6.1 | -0.16 | 5.9 | 6.0 | 0.17 | 3.8 | | 4.0 | 0.26 |
| PY mild dementia | 3.2 | 3.1 | 0.15 | 3.2 | 3.1 | -0.14 | 2.3 | | 2.3 | -0.01 |
| PY moderate dementia | 2.3 | 2.5 | -0.25 | 2.3 | 2.5 | 0.25 | 1.3 | | 1.5 | 0.23 |
| PY severe dementia | 0.4 | 0.5 | -0.06 | 0.4 | 0.5 | 0.06 | 0.2 | | 0.2 | 0.04 |
| PY dead | 18.4 | 18.7 | -0.27 | 8.6 | 8.8 | 0.26 | 1.5 | | 1.5 | 0.08 |
| Deaths | 99,944 | 99,947 | -3 | 94,051 | 94,710 | -659 | 39,257 | | 41,359 | -2,102 |
|  |  |  |  |  |  |  |  | |  |  |
| **BBM-CSF** | BBM-CSF | SoC | Diff | BBM-CSF | SoC | Diff | BBM-CSF | | SoC | Diff |
| PY alive | 11.6 | 11.3 | 0.27 | 11.4 | 11.2 | -0.26 | 8.5 | | 8.5 | -0.08 |
| PY nondementia | 5.6 | 5.2 | 0.44 | 5.6 | 5.1 | -0.43 | 4.8 | | 4.4 | -0.34 |
| PY dementia | 5.9 | 6.1 | -0.16 | 5.9 | 6.0 | 0.17 | 3.8 | | 4.0 | 0.26 |
| PY mild dementia | 3.2 | 3.1 | 0.15 | 3.2 | 3.1 | -0.14 | 2.3 | | 2.3 | -0.01 |
| PY moderate dementia | 2.3 | 2.5 | -0.25 | 2.3 | 2.5 | 0.25 | 1.3 | | 1.5 | 0.23 |
| PY severe dementia | 0.4 | 0.5 | -0.06 | 0.4 | 0.5 | 0.06 | 0.2 | | 0.2 | 0.04 |
| PY dead | 18.4 | 18.7 | -0.27 | 8.6 | 8.8 | 0.26 | 1.5 | | 1.5 | 0.08 |
| Deaths | 99,944 | 99,947 | -3 | 94,051 | 94,710 | -659 | 39,257 | | 41,359 | -2,102 |

PY: person years


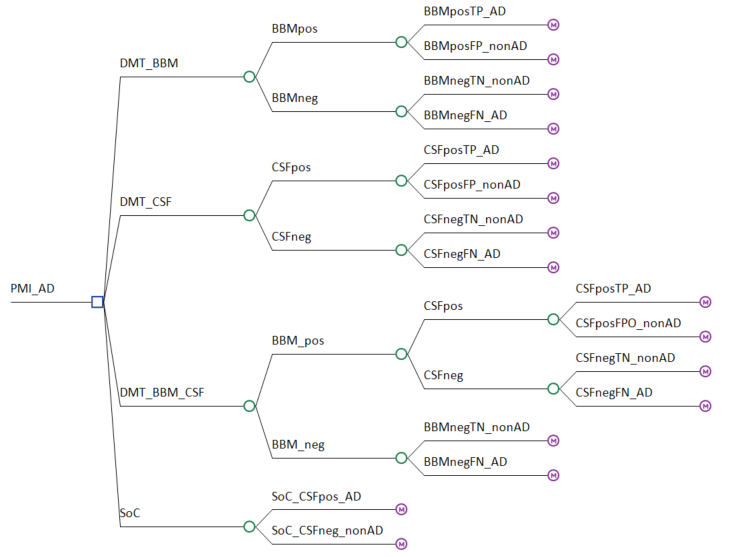


**Supplemental Figure 1.** Decision tree for the economic evaluations. The
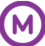
 indicates that at each such point, a Markov cohort is attached. Three sets of Markov transition probabilities are applied: 1) TP, 2) TN & FP, and 3) FN.


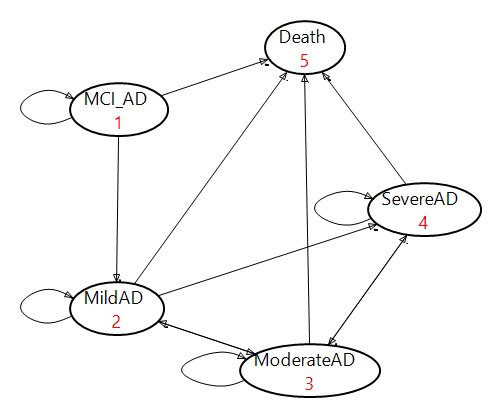


**Supplemental Figure 2.** The model structure of the Markov part of the model
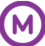
.

**References**

1. Wimo A, Handels R, Winblad B, et al. Quantifying and describing the natural history and costs of Alzheimer's disease and effects of hypothetical interventions. *J Alzheimers Dis* 2020; 75: 891-902.

2. Wimo A, Handels R, Winblad B, et al. Quantifying and describing the natural history and costs of Alzheimer's disease and effects of hypothetical interventions. *J Alzheimers Dis* 2020; 75: 891-902.

3. Handels R, Jonsson L, Garcia-Ptacek S, et al. Controlling for selective dropout in longitudinal dementia data: Application to the SveDem registry. *Alzheimers Dement* 2020; 16: 789-796.

4. Wimo A, Timón S, Bon J, et al. Costs of diagnosing early AD in three European memory clinic settings: Results from the PMI-AD project. *Int J Geriatr Psychiatry* 2024; 39: e6126.

5. Aye S, Handels R, Winblad B, et al. Optimising Alzheimer’s disease diagnosis and treatment: Assessing cost-utility of integrating blood biomarkers in clinical practice for disease-modifying treatment. *J Prev Alzheimers Dis* 2024; 11: 928-942.

6. Ekman M, Berg J, Wimo A, et al. Health utilities in mild cognitive impairment and dementia: a population study in Sweden. *Int J Geriatr Psychiatry* 2007; 22: 649-655.
